# Microstructure and Mechanical Properties of High-Strength AA6011 Aluminum Alloy Welding with Novel 4xxx Filler Metals

**DOI:** 10.3390/ma17020380

**Published:** 2024-01-12

**Authors:** Mohamed Ahmed, Mousa Javidani, Alexandre Maltais, X.-Grant Chen

**Affiliations:** 1 Department of Applied Science, University of Québec at Chicoutimi, Saguenay, QC G7H 2B1, Canada; mahmed2@etu.uqac.ca (M.A.); mjavidan@uqac.ca (M.J.); 2 Arvida Research and Development Center, Rio Tinto Aluminum, Saguenay, QC G7S 4K8, Canada; alexandre.maltais@riotinto.com

**Keywords:** high-strength aluminum alloy welding, AA6011 alloy, 4xxx filler metals, mechanical properties, impact toughness

## Abstract

Welding high-strength 6xxx aluminum alloys using a commercial ER4043 filler often results in inferior joint strength. This study investigated the effects of newly developed Al-Si-Mg filler metals with varying Mg (0.6–1.4 wt.%) and Mn (0.25–0.5 wt.%) contents on the microstructure evolution and mechanical performance of high-strength AA6011-T6 plates using gas metal arc welding. Two commercial fillers, ER4043 and ER4943, were used as references for comparison. The results revealed that increasing the Mg and Mn contents in the novel fillers resulted in sufficiently high alloying elements in the fusion zone (FZ), leading to higher microhardness. Under as-welded conditions, the weakest region of the joint was the heat-affected zone (HAZ). The joint strength was almost independent of the filler type and was controlled by the HAZ strength, measuring a UTS of 230 and 241 MPa for ER4043 and the other joints, respectively. The higher Mg contents in the novel fillers promoted the precipitation of a large volume fraction of fine β″-MgSi in the FZ during post-weld heat treatment (PWHT), resulting in superior strength and higher welding efficiency relative to the reference fillers. The optimal Mg content of the novel fillers was 0.6 wt.%. Increasing the Mn content of the filler metal had an insignificant effect. The FMg0.6 filler with 0.6% Mg achieved the best combination of strength (UTS of 410 MPa) and elongation (6.7%) as well as the highest welding efficiency (94%) after PWHT, among all of the fillers studied. However, the newly developed fillers adversely affected the impact toughness of the joints.

## 1. Introduction

Al-Mg-Si 6xxx alloys are widely used in the aerospace and automotive industries due to their high strength-to-weight ratio, good corrosion resistance, and weldability. However, the welding of 6xxx alloy parts using commercial Al-Si 4xxx filler metals often results in low welding strength, which is not acceptable for high-strength 6xxx alloy welding in many applications. The use of Al-Si 4xxx filler metals can be traced back to the 1940s. They have been widely used for the general-purpose welding of 6xxx alloys due to their low cost, high fluidity in the weld zone, and good resistance to weld cracks [1]. However, low welding strength is a persistent problem for high-strength 6xxx alloy joints [2]. Recent research has explored the development of new filler metals with improved mechanical properties [2,3,4] such as the use of nanoscale reinforcement particles [5] and advanced welding techniques such as friction stir welding [1]. However, the welding strength generally remains lower than the strength of the base metal (BM) [1,6,7].

The fusion welding process involves melting a part of the BM and filler metal together to form a weld bead or fusion zone (FZ). The composition of the FZ mainly depends on the composition of the BM and filler metal as well as the added ratio of these constituents, which is called the dilution ratio [8,9]. Furthermore, in the case of thick and multipass weldments, the dilution ratio calculation is complex because some parts of the previous pass are shared in the formation of the next pass [10]. The dilution is highest for single-pass welds on thin sheets with square joints, while it is lowest for filler welds with regular edge preparation and in the backup passes with thick BMs. Changes in the welding parameters and joint geometry can affect the fusion zone dilution and, hence, change the chemical composition of the weld zone [11]. Mousavi et al. [12] calculated the dilution ratio for different joint geometries and reported that a beveled joint (single V-groove) produced minimal BM dilution (40–60%), whereas butt joints resulted in high dilution (60–80%). Commercial ER4043 (Al-5Si) and ER4943 (Al-5Si-0.4Mg) wires are common filler metals used in Al welding. Due to the high Si content of these two fillers, the chemical composition of the FZ shifts toward an alloy regime that is less prone to hot cracking. However, because of the absence or insufficiency of Mg for Mg_2_Si precipitation strengthening in the weldments, the mechanical properties of high-strength 6xxx joints are often unsatisfactory. Therefore, the chemical composition of the FZ is the main factor affecting the metallurgical and mechanical properties of the weld and its susceptibility to cracking [13,14,15].

In addition to the mechanical properties, the Charpy impact test offers significant benefits such as determining the ductile-to-brittle transition and the relative toughness between different materials [16]. However, limited data are available in the literature on the impact toughness of Al alloys. Investigations of the impact properties have mainly focused on eutectic Al-Si cast alloys in the automotive industry [17,18]. Murali et al. [19] studied the effects of Mg and Fe impurities on the impact properties of hypoeutectic Al-Si-Mg cast alloys in the aircraft industry. They found that an increase in the Mg content from 0.32% to 0.65% caused a notable reduction of approximately 50% in the total absorption energy until fracture, which highlighted the possible embrittlement effect of Mg. The Fe intermetallics were investigated by Samuel et al. [20], who reported that a higher volume fraction of Fe intermetallics led to a reduction in impact toughness. Heat treatment was found to significantly enhance the impact energy of the as-cast 356 alloys, particularly when the iron content in the alloy was low [21]. Mosneaga et al. [22] found that the addition of Mn could enhance the impact toughness of the welded metal by 20–30%.

In our previous work [7], an AA6061-T6 thin sheet with a thickness of 2 mm was welded to the newly developed 4xxx fillers. The results showed that a high dilution ratio of approximately 56% produced an increase in the Mg content of the FZ, even if the Mg-free ER4043 filler was used. Consequently, the FZ became heat-treatable and exhibited a reasonably high tensile strength. However, when thick plates of high-strength AA6011 BM (YS > 400 MPa) were welded using multiple passes, the dilution ratio from the BM was low, and only a small amount of Mg from the BM could be transferred to the FZ. Consequently, the joint strength decreased depending on the amount of Mg transferred. In this study, four novel filler metals were designed with a Mg content of 0.6–1.4 wt.% and Mn content of 0.25–0.5 wt.%. High-strength AA6011-T6 plates with a thickness of 6 mm were welded to systematically investigate the effect of filler metals on the mechanical performance and microstructural evolution of the joints under as-weld and post-weld heat treatment (PWHT) conditions. ER4043 and ER4943, two reference fillers, were used for comparison with the newly developed fillers.

## 2. Materials and Methods

**Figure 1:**
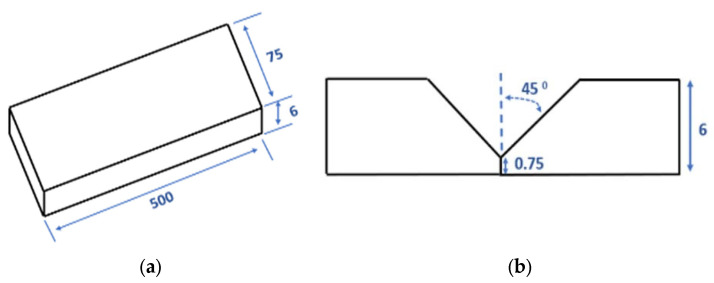
(**a**) Dimensions of the extruded BM and (**b**) single V–groove joint (mm).

The BM of the AA6011 alloy was direct chill cast and extruded into plates with a cross-section of 75 mm × 6 mm, which were then cut into a plate with a length of 500 mm and heat-treated in the T6 temper. The plates were welded using the gas metal arc welding (GMAW) technique. The filler wires used in this study were composed of four newly developed filler metals and two reference filler metals (commercial ER4043 and ER4943). The chemical compositions of the BM and filler metals were analyzed by the inductively coupled plasma technique, and the results are listed in Table 1. The process for producing new filler wires was reported in our previous study [7]. A Fronius Transpulse Synergic 5000-CMT mounted on a Motoman UP50N robot was used for the welding. Before welding, the plates were clamped in a butt-joint configuration with a single V-groove geometry, as shown in Figure 1. The welding parameters used in the two passes are listed in Table 2. A higher current for the first pass (163 A) was selected with the aim of achieving a greater melt penetration due to the groove geometry. The current of the second pass (110 A) necessitated reduced melt penetration and heat input [23]. Following the welding process, a part of the weld plates underwent post-weld heat treatment (PWHT) including a solution treatment at 550 °C for 1 h and water quenching, and natural aging for 24 h, followed by artificial aging at 180 °C for 8 h. The heat treatment was optimized to determine the mechanical properties of the BM during welding. 

**Figure 2:**
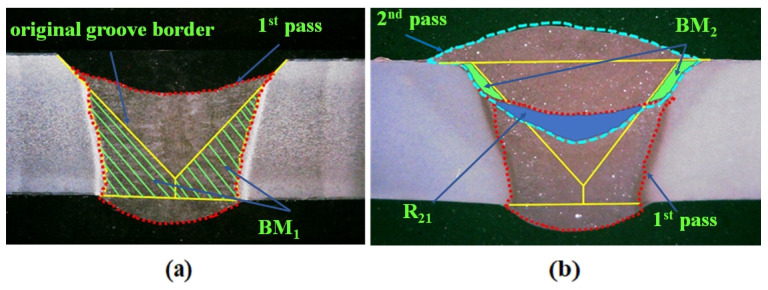
Macroview of a typical cross section joint for the dilution calculation. The areas for the (**a**) first pass and (**b**) second pass. R_21_ in (**b**) is the melted area of the first pass during the second pass.

The dilution ratios of the weld zone were calculated using the following equation [10]:

$$D_{n}=[{BM}_{n}+\sum_{j}^{n-1}R_{nj}{.D}_{j}]/A_{n}$$

where $D_{n}$ is the dilution with BM in pass *n*; *n* is the pass number; ${BM}_{n}$ is the melted BM area by pass number *n*; *j* is the number of previously deposited passes, $R_{nj}$ is the melted area from the previous pass *j* in pass *n*; $D_{j}$ is the dilution with BM in the weld zone for bead *j*; $A_{n}$ is the total area of the BM in pass *n* under the as-deposited condition. All the areas are illustrated in Figure 2. The calculated dilution ratios in the first and second passes are 43 ± 0.02% and 21 ± 0.06%, respectively. The chemical composition in each pass in the weld zone was calculated, and the results are listed in Table 3. 

**Figure 3:**
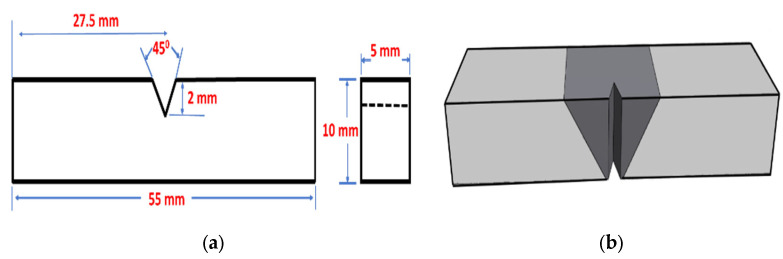
(**a**) Dimensions of the impact samples and their notches. (**b**) The notch position in the weld zone [17].

Microstructural analyses were performed using optical microscopy (OM), scanning electron microscopy (SEM), and transmission electron microscopy (TEM). The microhardness (HV) profiles were measured using an NG-1000 CCD microhardness tester with a 50-g load and 20-s dwell time. The HV measurements for each joint were obtained at two specific locations: the first location was positioned 1 mm from the bottom (representing the first pass), and the second location was located 1 mm from the upper surface (representing the second pass). Tensile tests were performed with an Instron 8801 servo-hydraulic machine under as-welded (AW) and PWHT conditions, using a crosshead speed of 1 mm/min at room temperature with standard subsize samples, according to [24]. For each filler type and condition, at least five tensile samples were tested, and the average and standard deviation values were calculated. The welding efficiency was calculated based on the formula of (UTS_joint_/UTS_BM_ × 100) [7]. Impact tests were conducted on the AW and PWHT samples to evaluate the impact toughness of the filler joints. Perpendicular to the weld bead, half-sized Charpy V-notch specimens with dimensions of 10 mm × 5 mm × 55 mm, notch root radius of 0.25 mm, notch depth of 2 mm, and notch flank angle of 45° were machined according to [25]. The notch in the HAZ was machined 8 mm from the center of the FZ. Figure 3 shows the geometries of the impact samples. An instrumented Charpy impact testing machine with a capacity of 150 J, an impact speed of 5.2 m/s, and a pendulum drop angle of 150° were used.

## 3. Results and Discussion

### 3.1. Microstructure

**Figure 4:**
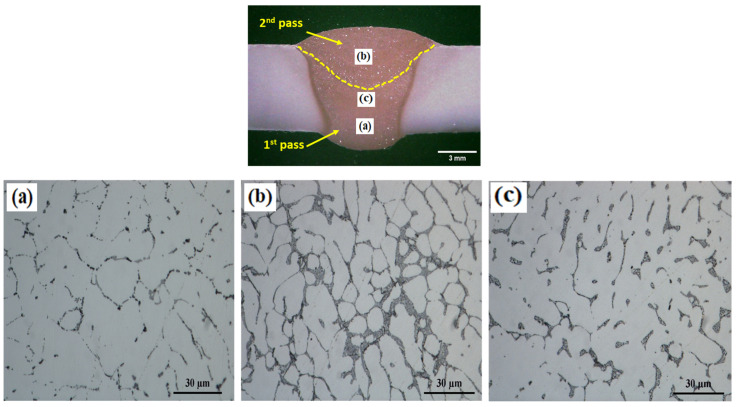
Optical microscopy of the different zones of the as-welded FMg1.4 joint. (**a**,**b**) The microstructure in the first and second passes, respectively. (**c**) The heat affected zone in the first pass by the heat input of the second pass.

Figure 4 shows the optical microstructure of the FMg1.4 joint, which is a typical example of a newly developed filler metal. Figure 4a displays the OM image of the first pass, which consists of α-Al dendrite cells/grains as the matrix and the surrounding eutectic Al-Si. In the second pass, the amount of eutectic Al-Si increased and the α-Al cells/grains coarsened (Figure 4b) due to lower BM dilution and a lower cooling rate relative to the first pass. Figure 4c shows the effect of the heat input to the second pass on the microstructure of the first pass. The heat introduced in the second pass resulted in the fragmentation of the eutectic Al-Si network and partial spheroidizing of eutectic Si particles in the transition zone of the first pass. 

**Figure 5:**
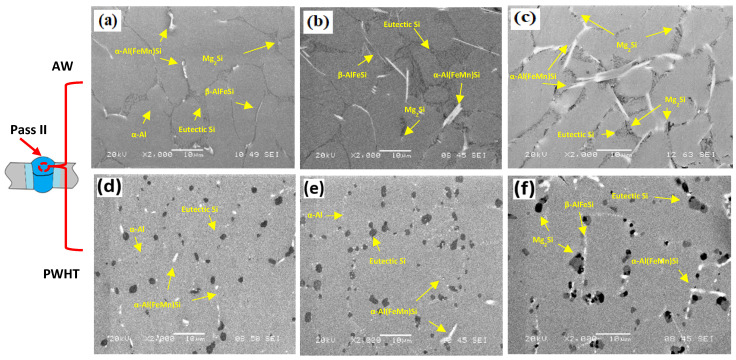
SEM microimages showing the microstructures of the second pass: (**a**) ER4043, (**b**) FMg0.6, and (**c**) Mg1.4Mn joints under the as-welded condition; (**d**) ER4043, (**e**) FMg0.6, and (**f**) Mg1.4Mn joints under the PWHT condition, respectively.

**Figure 6:**
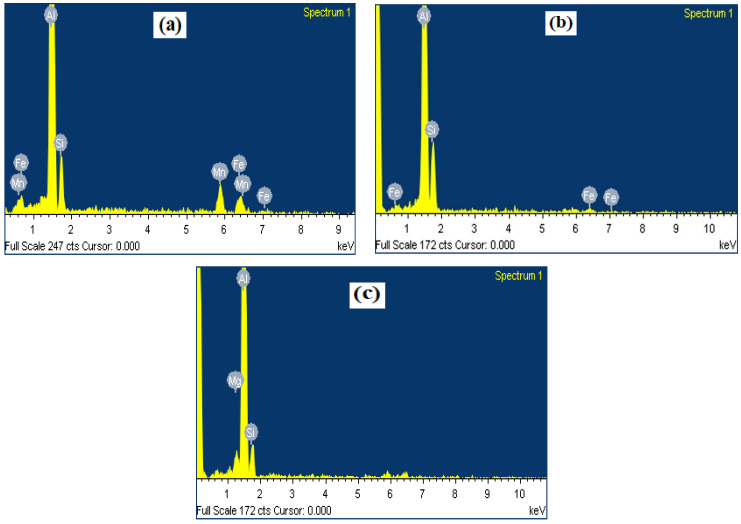
The typical SEM-EDS spectra representing the intermetallic phases (**a**) α-Al(FeMn)Si, (**b**) β-AlFeSi, and (**c**) Mg_2_Si in Figure 5.

Figure 5a–c shows the SEM micrographs of the as-welded FZs of the three filler metals. Attention was focused on the microstructures of the second pass as they revealed large differences between the joints using low-Mg reference fillers and the new high-Mg fillers. Generally, the microstructure was comprised of α-Al, eutectic Si, primary Mg_2_Si, α-Al(FeMn)Si, and β-AlFeSi intermetallics. All phases were identified based on their morphology and SEM-EDS analysis. The typical EDS spectra of three intermetallic phases (α-Al(FeMn)Si, β-AlFeSi and Mg_2_Si) are shown in Figure 6. As the Mn content increased in the new fillers including the FMg0.6 and Mg1.4Mn joints (Table 2), the plate-like β-AlFeSi began to transfer into α-Al(FeMn)Si [26]. In the Mg1.4Mn joint, the predominant Fe-rich intermetallic became α-Al(FeMn)Si (Figure 5c). Even in the absence of Mg in the ER4043 reference filler (Figure 5a), some Mg_2_Si particles were still observed in the FZ due to dilution from the BM. The volume fractions of the Mg_2_Si in the reference fillers ER4043 and ER4943 were 0.15 ± 0.08 and 0.43 ± 0.12%, respectively, whereas in the new fillers, the fractions of Mg_2_Si were significantly higher, being 0.75 ± 0.21% and 1.43 ± 0.25% in the fillers containing 0.6 and 1.4% of Mg, respectively. The volume fraction of Fe-rich intermetallics was dependent on the Mn content. The reference fillers had a volume fraction of 0.88 ± 0.24, whereas the fillers containing 0.25 and 0.5% Mn had volume fractions of 1.68 ± 0.32% and 2.31 ± 0.27%, respectively. 

Figure 5d–f shows the microstructures of the three filler metals under the PWHT condition. The heat treatment had a significant effect on the microstructure. The eutectic Si was spheriodized, and the β-AlFeSi intermetallic particles were partially fragmented [27]. This fragmentation is associated with the partial dissolution of Fe-rich intermetallics and their connected eutectic constituents such as Mg_2_Si [28,29,30]. Meanwhile, the α-Al(FeMn)Si particles were not affected by the PWHT because they are more stable than the β-AlFeSi particles, as shown in Figure 5f [29]. In the ER4043 and FMg0.6 samples, almost all of the Mg_2_Si particles were completely dissolved (Figure 5d,e), whereas undissolved primary Mg_2_Si particles remained in the Mg1.4Mn sample (Figure 5f) because the Mg content in this filler is higher than the maximum solubility of Mg at the solution treatment temperature of 550 °C, which is approximately 0.7 wt.% at this temperature [31].

**Figure 7:**
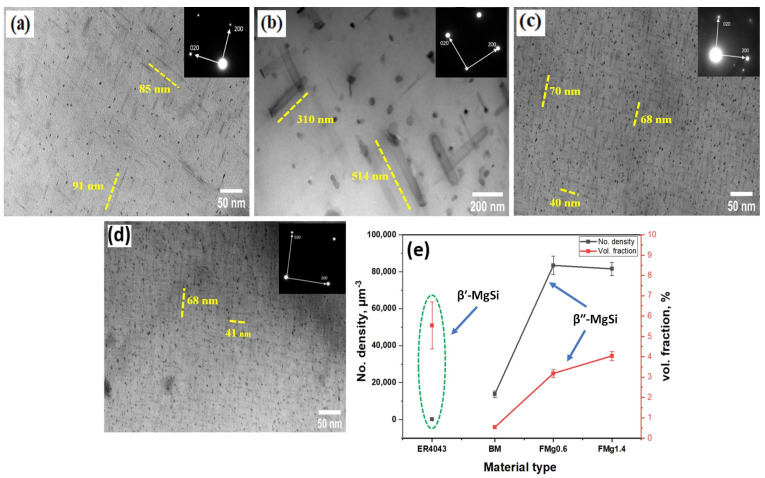
Bright-field TEM images of (**a**) β″ precipitates in the BM; (**b**) β′ precipitates in ER4043; β″ precipitates in (**c**) FMg0.6; (**d**) FMg1.4 joints. (**e**) Quantitative analysis of the precipitates.

TEM analysis was used to study the effect of PWHT on the precipitation of the joints, and the results are presented in Figure 7. The precipitates in all of the FZs were MgSi-type phases, as expected in AA6011 heat-treatable alloys. Based on the morphology and size, the precipitates in the BM, and all joints, except for the ER4043 joint, were identified as β″-MgSi. In the ER4043 joint, they were β′-MgSi [32]. The BM exhibited a lower number density of β″ (Figure 7a) compared to the FZs of the FMg0.6 and FMg1.4 joints, as shown in Figure 7c,d. This result is most likely attributed to the higher amount of Si in the FZs of the FMg0.6 and FMg1.4 joints, which promotes the formation of the coherent β″ precipitates [33]. The reason behind the formation of β′ in the FZ of ER4043 (Figure 7b) is the insufficient Mg content in the joint because the ER4043 filler itself contains almost no Mg. In alloys with low Mg content, β′ has been reported to precipitate at a lower temperature than β″ [34,35]. As the aging time was the same for all joints, the formation of β′ was promoted in the ER4043 sample. A quantitative analysis of the precipitates is shown in Figure 7e. The BM had a low number density and volume fraction of the β″. The number density and volume fraction of the β″ precipitates were comparable in the FMg0.6 and FMg1.4 joints, but their values were higher than the corresponding values in the BM. This implies that the Mg solutes dissolved in the low-Mg (FMg0.6) and high-Mg (FMg1.4) joints were mostly similar. As above-mentioned, not all of the primary Mg_2_Si in the FMg1.4 joint can be dissolved during PWHT. Therefore, the strengthening effect of β″ precipitates in both joints is expected to be similar. This result is consistent with the HV and tensile strength results discussed in the following sections.

### 3.2. Mechanical Properties

#### 3.2.1. Microhardness

**Figure 8:**
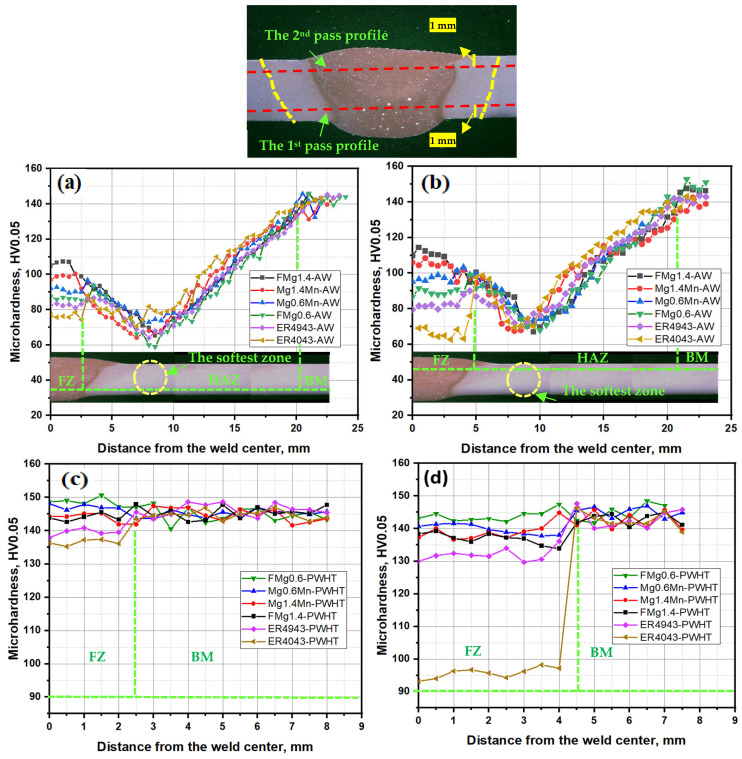
Hardness measurements in the joints for the (**a**) first and (**b**) second passes in the as-welded condition and (**c**) first and (**d**) second passes in the PWHT condition.

The as-welded HV profiles of the reference and newly developed fillers are illustrated in Figure 8a,b. The FZs of the new fillers had higher HV values than those of the reference fillers. Moreover, the second pass yielded higher HV values (90–115 HV, Figure 8b) than the first pass (86–107 HV, Figure 8a) for all new fillers. The ER4043 joint exhibited the lowest HV values among all of the filler metals used. However, it displayed a higher HV in the first pass than in the second pass (76 HV vs. 63 HV). This occurred because of the transfer of more Mg solutes from the BM in the first pass but less Mg in the second pass. The FZ of the ER4943 filler transferred similar HV values in both passes because the Mg content in both passes was within a similar range during dilution (Table 3). The Mg content in all of the fillers played a more predominant role in the microhardness profiles than the Mn content. At the same Mg content, a higher Mn content in the FZ resulted in a slightly higher HV [36]; however, the effect was relatively weak. The order of the average HV in the FZ in both passes was FMg1.4 > Mg1.4Mn > Mg0.6Mn > FMg0.6, which was determined based on the calculated Mg and Mn contents, as shown in Table 3. The softest region in the AW condition was the HAZ, which was positioned approximately 8 mm from the center of the FZ, with a total length of approximately 18 mm, as shown in Figure 8a,b. 

Figure 8c,d shows the HV profiles of the PWHT samples. The PWHT resulted in a significant increase in the HV values in both passes as well as in all fillers by promoting the precipitation strengthening arising from β″/β′ (Figure 7). The new fillers showed higher HV values than the two reference fillers. In the new fillers, the average HV values were 145 ± 5 HV in the first pass (Figure 8c) and 140 ± 5 HV in the second pass (Figure 8d). The ER4043 joint exhibited the lowest HV value for both passes, followed by the ER4943 joint. In particular, the HV of the ER4043 joint in the second pass decreased to 96 HV, which was much lower than that in the first pass. This reduction in HV can be attributed to the low Mg content in the FZ in the second pass and hence caused lower precipitation strengthening of coarse β′ (Figure 7b). In the new fillers, the differences in HV between the fillers and passes were quite small due to the limited Mg solubility and the similarity in the number densities of the precipitates, as shown in Figure 7e. Overall, PWHT was found to be effective in improving the HV of all of the fillers, and the new fillers showed a large improvement in HV because of the high Mg content in the FZ compared to the low BM dilution in the reference ER4043 filler. The HAZ disappeared after the PWHT due to the recovery of strength in this zone and the re-precipitation of the β″ strengthening phase in the matrix [7,37].

#### 3.2.2. Tensile Properties

**Figure 9:**
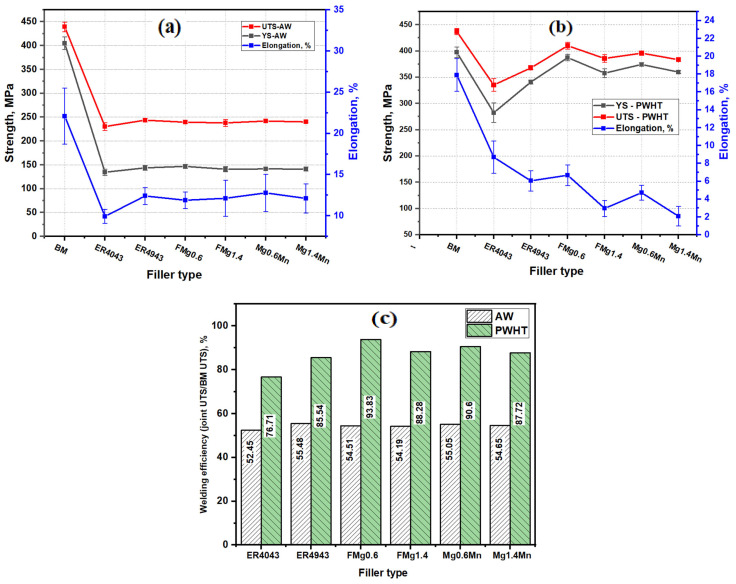
The tensile properties in the (**a**) as-welded and (**b**) PWHT conditions; (**c**) the welding efficiency in the as-welded and PWHT conditions.

The tensile properties of the as-welded joints are shown in Figure 9a. All of the tensile samples except for the ER4043 joint were fractured in the HAZ. The ER4043 joint was fractured in the FZ. The ER4043 joint presented the lowest YS and UTS of 134 ± 6 and 230 ± 8 MPa, respectively. This result is consistent with the HV results, as the lowest HV was observed for the ER4043 filler (Figure 8a,b). The ER4943 filler and new filler joints displayed similar YS and UTS values of 142 ± 4 and 241 ± 3 MPa, respectively. This can be attributed to the fact that the HAZ in all joints was the softest zone and had the minimum HV, which was also observed in our previous study on AA6061 welding [7,38]. The elongation of the as-weld joints exhibited a similar trend as for the tensile strength, with an average elongation of 12 ± 2%, except in the case of the ER4043 joint, which produced the lowest elongation of 9.5 ± 0.8%. The ER4043 filler had the lowest welding efficiency (52.5%) among all the fillers used. The average welding efficiency of all the as-welded joints was approximately 54.4%, which indicates that almost 45% of the AA6011-T6 strength was lost during the welding process (see Figure 9c). The joint strength in the as-welded condition represented the strength of the HAZs because all of the samples were fractured in this zone (except for the ER4043 joint). This implies that the actual strength of the FZs was higher than the obtained current strength. 

Figure 9b shows the tensile properties of the PWHT joints. The tensile strengths of all joints were consistent with the HV results, showing a significant improvement relative to the as-welded condition due to precipitation strengthening after PWHT. Fractures occurred in the FZs of all the welded joints. The ER4043 joint exhibited the lowest tensile strength (UTS of 335 ± 10 MPa), and the ER4943 joint showed greater tensile strength with a UTS of 373 ± 5 MPa due to the high Mg content in the filler. However, the tensile strengths of the two reference fillers were still lower than those of the new fillers due to the lower Mg content in the fillers relative to the new fillers. The average YS and UTS of the new fillers reached 370 ± 10 and 395 ± 12 MPa, respectively, which were still lower than those of the BM, although the number density and volume fraction of the β″ strengthening phase in the FZs of the new fillers were higher than those of the BM, as shown in Figure 7e. This is mostly due to the existence of welding defects such as porosity, which will be discussed in the fracture analysis section [39]. In addition, the tensile strengths of the four new fillers were similar, as these strengths depended on the number density and volume fraction of β″. According to the TEM results (Figure 7), the number density and volume fraction of the β″ precipitates in the low- and high-Mg fillers such as FMg0.6 and FMg1.4 were similar. Due to its low tensile strength, the ER4043 joint produced the highest elongation of 8.5 ± 2%. The elongation of the other fillers decreased considerably with the increasing Mg content [40]. The elongation of the ER4943 and FMg0.6 joints attained a similar value of 6.5 ± 1%. These results demonstrate that an excellent combination of strength and ductility was achieved using the FMg0.6 filler. With a further increase in Mg in the fillers such as in FMg1.4, or an increase in Mn in the fillers such as in Mg0.6Mn and Mg1.4Mn, the elongation decreased remarkably. The reasons behind this reduced ductility are attributed to (1) the undissolved brittle primary Mg_2_Si particles in the high-Mg filler and (2) the increase in the size and amount of α-Al(FeMn)Si intermetallic particles in the high-Mn fillers (Figure 5c,f). The welding efficiency increased from 76.7% for the ER4043 joint to 85.5% for the ER4943 joint. On the other hand, the average welding efficiency of the four newly developed fillers was 90.1%, whereas the FMg0.6 joint achieved the highest efficiency of 93.8% (Figure 9c).

#### 3.2.3. Fracture Analysis of Tensile Samples

**Figure 10:**
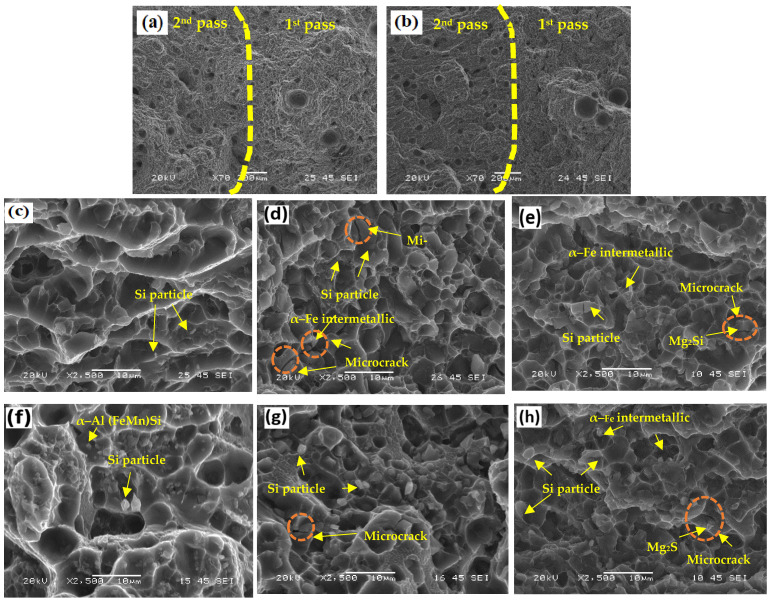
Fracture surfaces of the PWHT tensile samples. Porosity in the (**a**) ER4043 and (**b**) Mg1.4Mn joints. (**c**–**e**) First pass of the ER4043, FMg0.6, and Mg1.4Mn joints. (**f**–**h**) Second pass of the ER4043, FMg0.6, and Mg1.4Mn joints.

Figure 10 shows the fracture surfaces of the PWHT tensile samples. Figure 10a,b shows the pores formed during both passes in the ER4043 and Mg1.4Mn joints, respectively. A large number of pores were found in the second pass, whereas few but relatively large pores were observed in the first pass. This occurred because the heat input from the second pass resulted in gas release and caused the growth of pre-existing pores in the first pass [40,41,42]. The fracture surfaces of the three joints (ER4043, FMg0.6, and Mg1.4Mn) in the first pass are illustrated in Figure 10c–e. The fracture surface of the ER4043 joint showed large and deep dimples, indicating a ductile fracture. The fracture surfaces of the FMg0.6 and Mg1.4Mn joints exhibited honeycomb-like shallow dimples, suggesting a more brittle fracture than that of the ER4043 joint. The dimples of the Mg1.4Mn joint were smaller and shallower than those of the FMg0.6 joint, indicating its more brittle nature. Broken particles, primarily Si and α-Fe intermetallic particles, were observed at the bottom of the dimples in the ER4043 joint and the joints with low-Mg content fillers (e.g., FMg0.6 and Mg0.6Mn). In joints with high-Mg content fillers (e.g., FMg1.4 and Mg1.4Mn), the broken α-Fe intermetallic particles with the undissolved primary Mg_2_Si particles were observed in the bottom of the dimples. Both α-Fe intermetallic and primary Mg_2_Si particles were mainly distributed along interdendritic boundaries. Moreover, microcracks can easily initiate and propagate along these brittle particles [43,44]. 

The fracture surfaces of the second pass under the PWHT condition are shown in Figure 10f–h. Similar to the first pass, the ER4043 joint had a fracture surface, indicating a more ductile fracture in the second pass, whereas the dimples on the new filler joints were smaller and shallower than those of the ER4043 joint. The FMg0.6 joint showed relatively larger dimples, illustrating better ductility than the Mg1.4Mn joints, which is consistent with the measured elongations (see Figure 9). Due to the high Si content in the second pass, a larger number of fractured Si particles were observed than in the first pass.

#### 3.2.4. Charpy Impact Toughness

**Figure 11:**
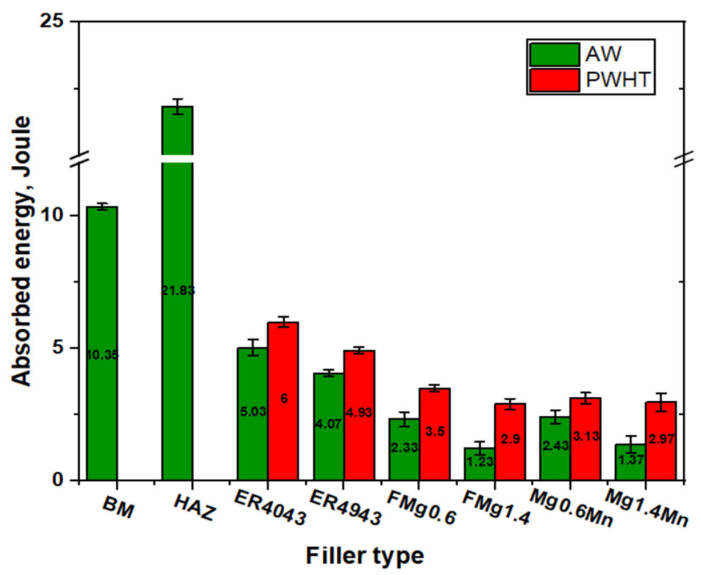
Impact toughness of the BM, HAV, and FZ of all joints under as-welded and PWHT conditions.

Figure 11 shows the impact toughness of the BM, HAZ, and FZ of the joints under as-welded and PWHT conditions. The HAZ exhibited the highest impact toughness (21.83 J), representing the highest absorbed energy relative to the FZ of all the joints. The impact toughness in the HAZ can be attributed to the effect of the welding heat input, which resulted in the dissolution and coarsening of the β″ precipitates and the HAZ becoming the softest region in the joints (Figure 8a,b), which could absorb the impact energy. The BM presented the second highest value of impact toughness of 10.35 J due to the absence of macrodefects such as porosity in the extruded plate, and the best ductility compared to the welded joints (Figure 9a). Under the as-weld condition, the ER4043 and ER4943 joints showed higher values of impact toughness (5.03 and 4.07 J) compared to those of the new filler joints. The joints with the fillers containing 0.6% Mg (FMg0.6 and Mg0.6Mn) had an impact toughness of 2.38 ± 0.07 J, whereas the joints with the high-Mg fillers containing 1.4% Mg (FMg1.4 and Mg1.4Mn) could absorb the impact energy up to 1.3 ± 0.01 J. The decrease in the impact toughness with increasing Mg contents in the new fillers is mainly attributed to (1) the increased amount of primary Mg_2_Si particles (Figure 5b,c), which can act as a stress riser and promote the formation of microcracks [45], and (2) the increasing Mg solutes in the matrix, thereby retarding the dislocation movement. In the case of the impact test, the dislocations had no time to override the Mg effect due to the high strain rate; hence, less impact energy was absorbed.

The joints subjected to PWHT exhibited a moderate improvement in impact toughness compared with those subjected to the as-welded condition. This improvement can be attributed to a few factors including the spheroidization of eutectic Si particles and the dissolution of primary Mg_2_Si particles during solution treatment (Figure 5d–f) [21]. Under the PWHT condition, the ER4043 and ER4943 joints achieved impact toughness values of 6 ± 0.7 and 4.93 ± 0.35 J, respectively. Despite the improved impact toughness of the PWHT samples, the joints with the new fillers still exhibited lower toughness than the joints with the two reference fillers. This difference in impact toughness is mostly due to the precipitation of the high number density of the β″ strengthening phase in the joints with the new fillers, resulting in high tensile strength but relatively low elongation (the trade-off between strength and ductility) [46,47].

#### 3.2.5. Fracture Analysis of Impact Samples

**Figure 12:**
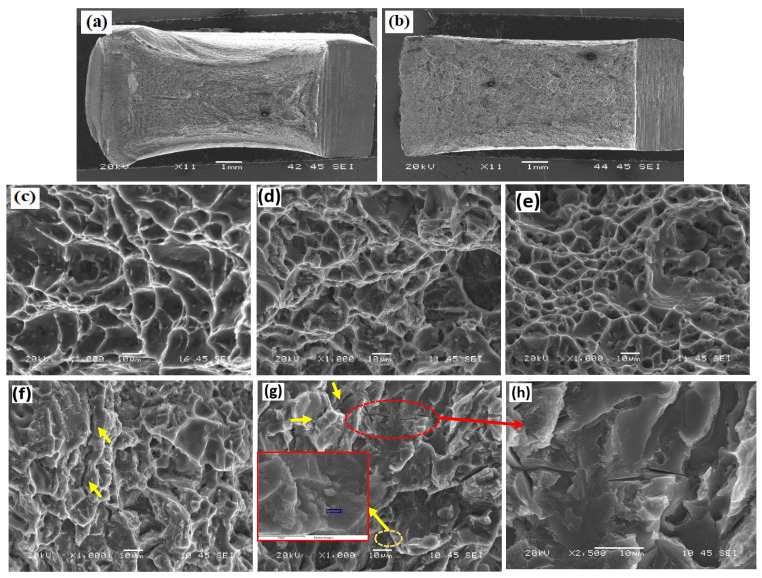
Fracture surfaces of the impact samples under the as-welded condition: macroview of the (**a**) HAZ and (**b**) ER4043 joint in the first pass. Microview of the (**c**) HAZ, (**d**) and (**e**) ER4043 joint in the second pass. Microview of the FMg1.4 joint in the (**f**) first and (**g**) second passes, and (**h**) secondary cracks starting from the intermetallic phases.

Figure 12 shows the fracture surfaces of the as-welded impact samples. A higher level of plastic deformation was observed in the HAZ than in the ER4043 or any other joint. A typical example of the macroview of the HAZ and ER4043 fractures is shown in Figure 12a,b. The HAZ exhibited large and deep dimples, showing significant deformation, and high impact energy was absorbed (Figure 12c) [48]. The fracture surfaces of the ER4043 joint still exhibited ductile fractures in the first and second passes, as shown in Figure 12d,e. 

Figure 12f,g depicts the fracture surfaces of the high-Mg filler (FMg1.4) in the first and second passes, respectively. In both passes, the cleavage facets became the dominant feature of the fracture, with only a few small dimples. This brittle fracture in the FMg1.4 joint was caused by the high amount of primary Mg_2_Si and Fe-rich intermetallic particles, which resulted in low ductility and premature failure. Furthermore, secondary cracks were observed in the fracture surfaces of the FMg1.4 joint in both passes (see the yellow arrows in Figure 12f,g and the enlarged images in Figure 12h), which mainly started from the primary Mg_2_Si and Fe-rich intermetallic particles.

### 3.3. Comparison of the Welding Efficiency of Various Al-Mg-Si Alloys

Table 4 shows a comparison of the welding efficiency of the newly developed fillers with previously reported fusion welding efficiencies of various Al-Mg-Si alloys under AW and PWHT conditions. The welding efficiency under the AW condition when the commercial fillers ER4043 or ER4943 are used for various Al-Mg-Si 6xxx-based materials with different thicknesses ranges from 33.6 to 69.7%, with an average value of 54.6%. The average efficiency of the different 6xxx alloy welds was consistent with that of the newly developed fillers (Figure 9c). This suggests that the welding efficiency under the AW condition was almost independent of the welding technique (tungsten inert gas (TIG) vs. gas metal arc welding (GMAW)), the base materials, and their thickness. The fractures of the tensile samples commonly occurred in the FZ or HAZ. This is primarily attributed to the occurrence of weldment flaws or defects in the FZs and an insufficient strengthening source in the FZs (using ER4043/4943 fillers), which resulted in a considerable reduction in the FZ strength. When the FZ became stronger such as when using the new fillers in this work, the weakest region of the welding was transferred to the HAZ, where the precipitation strengthening of the 6xxx alloys was largely demolished. 

Generally, the PWHT weldments exhibited much better welding efficiency than the AW weldments. This is due to the formation of the β″/β′ strengthening phase in the FZ and the strength recovery of the HAZ. The fracture of the tensile samples for all 6xxx alloys occurred only in the FZ because the heat treatment removed the HAZ. Under the PWHT condition, the welding efficiency when the ER4043/493 fillers were used for various 6xxx alloys ranged from 51.5 to 85.5%, with an average value of 70.7%. The average welding efficiency of the newly developed fillers with the base material AA6011 was 90.1% (Figure 9c), whereas the new FMg0.6 filler exhibited the highest efficiency of 93.8% among all the welded materials listed in Table 4. This is primarily attributed to the higher Mg content in the new filler metals and, hence, the higher precipitation strengthening effect in the FZ compared to the ER4043/493 reference fillers (Table 3).

### 3.4. Discussion

In the present work, it was confirmed that using a commercial ER4043 filler (free of Mg) to weld a high-strength AA6011 alloy produced inferior strength and low welding efficiency because of the low dilution and insufficient Mg in the weldments. ER4943 filler with Mg increased to 0.4 wt.% improved the strength and welding efficiency. Further increasing the Mg and Mn contents in the newly developed fillers led to high dilution and an increase in the alloying elements (mainly Mg and Mn) in the FZ, resulting in higher solid solution strengthening and higher microhardness in the FZ under the AW condition. However, the softest region of the joint in the AW condition was the HAZ, because the highest amount of β″-MgSi strengthening precipitates were dissolved in the HAZ due to the high welding temperature [7]. The joint strength under AW conditions was controlled by the strength of the HAZ (Figure 9a). Therefore, the new fillers with high Mg and Mn contents did not show a significant benefit under AW conditions in terms of strength and welding efficiency compared to the two reference fillers.

As the newly developed fillers provided sufficient Mg in the FZ, a large volume fraction of fine β″-MgSi was precipitated in the FZ during PWHT (Figure 7), resulting in superior strength and higher welding efficiency compared to the reference fillers (Figure 9b,c). The FMg0.6 filler, containing 0.6 wt.% Mg, achieved the best combination of strength and elongation and the highest welding efficiency among the four new fillers studied. Upon increasing the Mg content to 1.4 wt.% (such as in FMg1.4 and Mg1.4Mn fillers), the tensile strength remained at a similar level because the primary Mg_2_Si could not fully dissolve in the FZ during the solution treatment due to the Mg solubility limit (Figure 5f). However, the elongation decreased remarkably for the higher-Mg fillers. The increase in Mn content in the novel fillers at the same Mg content (Mg0.6Mn and Mg1.4Mn fillers) did not enhance the strength of the joints but rather reduced the elongation. Therefore, the Mg level in the filler metal plays a predominant role in improving the tensile strength and welding efficiency of high-strength AA6011 joints.

## 4. Conclusions

1. During the welding of thick plates of a high-strength AA6011 alloy using multiple passes, increasing the Mg and Mn contents of the Al-Si-Mg 4xxx filler metals resulted in sufficiently higher alloying elements in the FZ compared to the ER4043/ER4943 reference fillers, resulting in higher microhardness in the weldments.
2. Under the AW condition, the weakest region of the joint was the HAZ because the highest amount of β″-MgSi strengthening precipitates in the AA6011 alloy were dissolved. The joint strength was almost independent of the filler type and was controlled by the HAZ strength. The average welding efficiency of the as-welded joints was 54.4%.
3. The higher Mg contents in the newly developed fillers promoted the precipitation of a large volume fraction of fine β″-MgSi in the FZ during PWHT, resulting in superior strength and higher welding efficiency relative to the reference fillers. The Mg in the filler metals played a predominant role in improving the tensile strength and welding efficiency of the high-strength AA6011 joints.
4. The optimal Mg content of the filler metals was 0.6 wt.%. Upon further increasing the Mg content, the tensile strength remained at the same level, but the elongation decreased significantly. The novel fillers with 0.6% Mg content exhibited a complete dissolution of Mg_2_Si particles after PWHT, whereas the fillers with 1.4% Mg content retained undissolved Mg2Si in the matrix due to the Mg solubility limit. The increased Mn content in the filler metals had an insignificant effect on joint strength but caused a reduction in elongation.
5. The novel FMg0.6 filler containing 0.6% Mg achieved the best combination of strength and elongation after PWHT among all of the filler metals studied. In addition, the FMg0.6 filler exhibited the highest welding efficiency of 94% when comparing the data collected for all the welded Al-Mg-Si 6xxx alloys.
6. The newly developed fillers adversely affected the impact toughness of the joints under both AW and PWHT conditions. After performing the PWHT, the impact toughness of the novel fillers improved but was still lower than that of the reference fillers.

## Figures and Tables

**Table 1:** Chemical compositions of the base metal and filler metals (wt.%).

	Si	Mg	Mn	Fe	Cu	Ti
BM (6011-T6)	0.92	0.75	0.45	0.17	0.64	0.028
ER4043	4.97	0.024	0.02	0.16	-	0.108
ER4943	5.51	0.395	0.02	0.17	-	0.108
FMg0.6	6.23	0.6	0.23	0.14	0.011	0.103
FMg1.4	6.4	1.4	0.28	0.18	0.012	0.103
Mg0.6Mn	6.2	0.61	0.5	0.14	0.01	0.103
Mg1.4Mn	6.25	1.43	0.48	0.15	0.012	0.106

**Table 2:** The welding parameters used.

	Current (A)	Voltage (V)	Travel Speed (m/min)	Wire Feed Speed (m/min)
1st Pass	163 ± 4	21.5 ± 0.5	0.5	4.5 ± 0.3
2nd Pass	110 ± 5	22 ± 0.7	0.27	3.2

**Table 3:** The calculated chemical compositions in the first and second passes (wt.%).

		Si	Mg	Mn	Fe	Cu
First pass	ER4043	3.25	0.34	0.21	0.17	0.29
	ER4943	3.63	0.55	0.23	0.17	0.28
	FMg0.6	3.51	0.67	0.32	0.16	0.27
	FMg1.4	3.39	1.12	0.35	0.18	0.30
	Mg0.6Mn	3.83	0.68	0.47	0.16	0.31
	Mg1.4Mn	3.59	0.82	0.28	0.09	0.28
Second pass	ER4043	4.22	0.16	0.10	0.16	0.12
	ER4943	5.21	0.46	0.24	0.16	0.16
	FMg0.6	5.26	0.63	0.27	0.15	0.11
	FMg1.4	4.52	1.07	0.37	0.18	0.15
	Mg0.6Mn	4.44	0.66	0.48	0.15	0.13
	Mg1.4Mn	4.40	1.08	0.39	0.13	0.09

**Table 4:** Comparison of the welding efficiency for various Al-Mg-Si alloy fusion welding in both AW and PWHT conditions.

	No.	BM (Thickness)	Filler	Technique	Efficiency, %	Fracture	Reference
AW	1	6061 (6 mm)	4043	TIG	33.6	FZ	[49]
	2	6061 (3 mm)	4943	TIG	58.1	FZ	[5]
	3	6082 (3 mm)	4043	TIG	48.3	FZ	[4]
	4	6082 (3 mm)	4043 (0.35Ti)	TIG	57.1	FZ	[4]
	5	6082 (4 mm)	4043	TIG	69.7	HAZ	[50]
	6	6061 (6.35 mm)	4043	GMAW	53.3	FZ	[51]
	7	6061 (9 mm)	4043	GMAW	54.4	HAZ	[37]
	8	6061(9.5 mm)	4043	GMAW	58.7	FZ	[52]
	9	6061 (12.7 mm)	4043	GMAW	56.1	FZ	[53]
	10	6011 (6 mm)	4043	GMAW	56.1	HAZ	This work
	11	6011 (6 mm)	4943	GMAW	55.5	HAZ	This work
	12	6011 (6 mm)	Mg1.4Mn	GMAW	54.7	HAZ	This work
	13	6011 (6 mm)	FMg0.6	GMAW	54.2	HAZ	This work
PWHT	1	6061 (2.5 mm)	4043	TIG	74.2	FZ	[54]
	2	6061 (6.36 mm)	4043	TIG	51.5	FZ	[55]
	3	6082 (3 mm)	4043 (0.35Ti)	TIG	61.5	FZ	[4]
	4	6082 (12 mm)	4043	TIG	64.3	FZ	[56]
	5	6061 (3 mm)	4043	GMAW	76.0	FZ	[57]
	6	6061 (6.35 mm)	4043	GMAW	85.1	FZ	[51]
	7	6061 (9 mm)	4043	GMAW	53.0	FZ	[37]
	8	6061 (12.7 mm)	4043	GMAW	62.3	FZ	[53]
	9	6005 (3.2 mm)	4043	GMAW	80.1	FZ	[58]
	10	6063 (6.4 mm)	4043	GMAW	78.1	FZ	[58]
	11	6011 (6 mm)	4043	GMAW	76.7	FZ	This work
	12	6011 (6 mm)	4943	GMAW	85.5	FZ	This work
	13	6011 (6 mm)	Mg1.4Mn	GMAW	87.7	FZ	This work
	14	6011 (6 mm)	FMg0.6	GMAW	93.8	FZ	This work

## Data Availability

Supporting data could be made available upon reasonable request.

## References

[1] Mishra R.S., Ma Z.Y. (2005). Friction stir welding and processing. Mater. Sci. Eng. R Rep..

[2] Anderson T. (2013). A New Development in Aluminum Welding Wire: Alloy 4943. Weld. J..

[3] Babu N.K., Talari M.K., Pan D., Sun Z., Wei J., Sivaprasad K. (2012). Microstructural characterization and grain refinement of AA6082 gas tungsten arc welds by scandium modified fillers. Mater. Chem. Phys..

[4] Babu N.K., Talari M.K., Dayou P., Zheng S., Jun W. (2012). Influence of titanium–boron additions on grain refinement of AA6082 gas tungsten arc welds. Mater. Des..

[5] Bethea J.F. (2019). Gas Tungsten Arc Welding of Aluminum Alloys with Nanocomposite 4943 Filler Material. Master’s Thesis.

[6] Norman A.F., Birley S.S., Prangnell P.B. (2003). Development of new high strength Al–Sc filler wires for fusion welding 7000 series aluminium aerospace alloys. Sci. Technol. Weld. Join..

[7] Ahmed M., Javidani M., Mirakhorli F., Maltais A., Chen X.G. (2023). Developing High-Strength Al-Si-Mg Filler Metals for Aluminum Fusion Welding. J. Mater. Eng. Perform..

[8] Francis J.A., Bednarz B., Bee J.V. (2002). Prediction of steady state dilution in multipass hardfacing overlays deposited by self shielded flux cored arc welding. Sci. Technol. Weld. Join..

[9] Sun Y.L., Hamelin C.J., Flint T.F., Vasileiou A.N. (2019). Prediction of Dilution and Its Impact on the Metallurgical and Mechanical Behavior of a Multipass Steel Weldment. J. Press. Vessel. Technol..

[10] Vahid H., Kjell H., Leif K. (2020). Bead by bead study of a multipass shielded metal arc-welded super-duplex stainless steel. World Weld..

[11] Shahi A.S., Sunil P. (2008). Modelling of the effects of welding conditions on dilution of stainless steel claddings produced by gas metal arc welding procedures. Mater. Process. Technol..

[12] Mousavi M.G., Cross C.E., Grong Ø., Hval M. (1997). Controlling weld metal dilution for optimised weld performance in aluminium. Sci. Technol. Weld. Join..

[13] Coniglio N., Cross C.E., Michael T., Lammers M. (2008). Defining a critical weld dilution to avoid solidification cracking in aluminum. Weld. J..

[14] Ramanaiah N., Balakrishna B., Rao K.P. (2012). Effect of Modified AA5356 Filler on Corrosion Behavior of AA6061 Alloy GTA Welds. Int. J. Adv. Manuf. Technol..

[15] Ramanaiah N., Rao K.P. (2013). Effect of modified AA4043 filler on corrosion behavior of AA6061 alloy GTA welds. Int. J. Adv. Manuf. Technol..

[16] Schmitt W., Sun D.-Z., Böhme W. (1994). Evaluation of fracture toughness based on results of instrumented Charpy tests. Int. J. Press. Vessel. Pip..

[17] Li Z., Samuel A.M., Samuel F.H., Ravindran C. (2004). Parameters controlling the performance of AA319-type alloys: Part II. Impact properties and fractography. Mater. Sci. Eng. A.

[18] Basavakumar K., Mukunda P., Chakraborty M. (2008). Impact toughness in Al–12Si and Al–12Si–3Cu cast alloys—Part 1: Effect of process variables and microstructure. Int. J. Impact Eng..

[19] Murali S., Raman K.S., Murthy K.S.S. (1992). Effect of magnesium, iron (impurity) and solidification rates on the fracture toughness of Al7Si0.3Mg casting alloy. Mater. Sci. Eng. A.

[20] Abuhasel K.A., Ibrahim M.F.A., Elgallad E.M., Samuel F.H. (2016). On the impact toughness of Al–Si cast alloys. Mater. Des..

[21] Elsebaie O., Samuel A., Samuel F. (2011). Effects of Sr-modification, iron-based intermetallics and aging treatment on the impact toughness of 356 Al–Si–Mg alloy. J. Mater. Sci..

[22] Mosneaga V., Mizutani T., Kobayashi T., Toda H. (2002). Impact toughness of weldments in Al-Mg-Si alloys. Mater. Trans..

[23] Brungraber R.J., Nelson F.G. (1973). Effect of welding variables on aluminum alloy weldments. Weld. J..

[24] (2023). Standard Test Methods for Tension Testing Wrought and Cast Aluminum- and Magnesium-Alloy Products.

[25] (2023). Standard Test Methods for Notched Bar Impact Testing of Metallic Materials.

[26] Seifeddine S., Svensson I.L. (2009). The influence of Fe and Mn content and cooling rate on the microstructure and mechanical properties of A380-die casting alloys. Metall. Sci. Technol..

[27] Beroual S., Boumerzoug Z., Paillard P., Borjon-Piron Y. (2019). Effects of heat treatment and addition of small amounts of Cu and Mg on the microstructure and mechanical properties of Al-Si-Cu and Al-Si-Mg cast alloys. J. Alloys Compd..

[28] Moustafa M.A. (2009). Effect of iron content on the formation of β-Al_5_FeSi and porosity in Al–Si eutectic alloys. Mater. Process. Technol..

[29] Kumar S., Grant P.S., O’Reilly K.A.Q. (2012). Fe bearing intermetallic phase formation in a wrought Al–Mg–Si alloy. Trans. Indian Inst. Met..

[30] Liu Y.L., Kang S.B. (1997). The solidification process of Al–Mg–Si alloys. J. Mater. Sci..

[31] ASM-International (1991). Heat Treating—Heat Treating of Aluminum Alloys.

[32] Vissers R., Huis M.A.V., Jansen J., Zandbergen H.W. (2007). The crystal structure of the β′ phase in Al–Mg–Si alloys. Acta Mater..

[33] Gupta A.K., Lloyd D.J., Court S.A. (2001). Precipitation hardening in Al–Mg–Si alloys with and without excess Si. Mater. Sci. Eng. A.

[34] Wang Q.G., Davidson C.J. (2001). Solidification and precipitation behaviour of Al-Si-Mg casting alloys. J. Mater. Sci..

[35] Girelli L., Tocci M., Gelfi M., Pola A. (2019). Study of heat treatment parameters for additively manufactured AlSi10Mg in comparison with corresponding cast alloy. Mater. Sci. Eng. A.

[36] Nam S.W., Lee D.H. (2000). The effect of Mn on the mechanical behavior of Al alloys. Met. Mater..

[37] Pérez J.S., Ambriz R.R., López F.F.C. (2016). Recovery of mechanical properties of a 6061-T6 aluminum weld by heat treatment after welding. Metall. Mater. Trans. A.

[38] Ahmed M., Javidani M., Maltais A., Chen X.-G. (2023). Welding of AA6061-T6 Sheets Using High-Strength 4xxx Fillers: Effect of Mg on Mechanical and Fatigue Properties. Materials.

[39] Wang L., Liu Y., Yang C., Gaos M. (2021). Study of porosity suppression in oscillating laser-MIG hybrid welding of AA6082 aluminum alloy. J. Mater. Process. Technol..

[40] Pedersen L., Arnberg L. (2001). The effect of solution heat treatment and quenching rates on mechanical properties and microstructures in AlSiMg foundry alloys. Metall. Mater. Trans. A.

[41] Fahlström K., Blackburn J., Karlsson L. (2018). Low Porosity in Cast Magnesium Welds by Advanced Laser Twin-Spot Welding. Mater. Sci. Appl..

[42] Fahlström K., Blackburn J., Karlsson L. (2018). Effect of Laser Welding Parameters on Porosity of Weldsin Cast Magnesium Alloy AM50. Mod. Approaches Mater. Sci..

[43] Lin B., He X., Xu R., Zhao Y., Lu Y. (2023). Evolution of iron-rich intermetallics and its effect on the mechanical properties of Al–Cu–Mn–Fe–Si alloys after thermal exposure and high-temperature tensile testing. J. Mater. Res. Technol..

[44] Ibrahim M.F., Alkahtani S.A., Abuhasell K.A., Samuel F.H. (2015). Effect of intermetallics on the microstructure and tensile properties of aluminum based alloys: Role of Sr, Mg and Be addition. Mater. Des..

[45] Tocci M., Donnini R., Angella G., Pola A.J.M.C. (2017). Effect of Cr and Mn addition and heat treatment on AlSi_3_Mg casting alloy. Mater. Charact..

[46] Zhang D.L., Zheng L.H., StJohn D.H. (2002). Effect of a short solution treatment time on microstructure and mechanical properties of modified Al–7wt.% Si–0.3 wt.% Mg alloy. J. Light Met..

[47] Merlin M., Timelli G., Bonollo F., Garagnani G.L. (2009). Impact behaviour of A356 alloy for low-pressure die casting automotive wheels. J. Mater. Process. Technol..

[48] Ambriz R.R., Jaramillo D., Garcia C. (2016). Fracture energy evaluation on 7075-T651 aluminum alloy welds determined by instrumented impact pendulum. Trans. Nonferrous Met. Soc. China.

[49] Rojas H., Molina A., Valdez S., Campillo B. (2020). The impact of heat input on the microstructures, fatigue behaviors, and stress lives of TIG-welded 6061-T6 alloy joints. Mater. Res. Express.

[50] Ericsson M., Sandström R. (2003). Influence of welding speed on the fatigue of friction stir welds, and comparison with MIG and TIG. Int. J. Fatigue.

[51] Dewan M.W., Wahab M.A., Okeil A.M. (2015). Influence of weld defects and postweld heat treatment of gas tungsten arc-welded AA-6061-T651 aluminum alloy. J. Manuf. Sci. Eng..

[52] Ambriz R.R., Mesmacque G., Ruiz A., Amrouche A. (2010). Effect of the welding profile generated by the modified indirect electric arc technique on the fatigue behavior of 6061-T6 aluminum alloy. Mater. Sci. Eng. A.

[53] Ambriz R.R., Barrera G., García R., López V.H. (2010). The microstructure and mechanical strength of Al-6061-T6 GMA welds obtained with the modified indirect electric arc joint. Mater. Des..

[54] Haryadi G.D., Dewa R.T., Ekaputra I.M.W., Suprihanto A. (2020). Investigation of post-weld heat treatment (T6) and welding orientation on the strength of TIG-welded AL6061. Open Eng..

[55] Dewan M., Liang J., Wahab M., Okeil A. (2013). Post-weld residual stresses and heat treatments of gas tungsten arc welded aluminum alloy AA6061-T651. World J. Eng..

[56] Wang B., Xue S., Ma C., Wang J., Lin Z. (2017). Effects of porosity, heat input and post-weld heat treatment on the microstructure and mechanical properties of TIG welded joints of AA6082-T6. Metals.

[57] Ramaswamy A., Malarvizhi S., Balasubramanian V. (2020). Post-weld heat treatment effects on the tensile properties of cold metal arc welded AA 6061-T6 aluminum joints. Mater. Test..

[58] Menzemer C.C., Hilty E., Morrison S., Minor R., Srivatsan T.S. (2016). Influence of post weld heat treatment on strength of three aluminum alloys used in light poles. Metals.

